# Management of type 2 diabetes mellitus using Yoga: Implications for immunological and autonomic dysfunction

**DOI:** 10.6026/973206300214777

**Published:** 2025-12-15

**Authors:** Rukadikar C.A, Geetha M, Dixit A.M, Renuka K, Vinoth R, Nishad S, Rukadikar A.R, Singh M.K

**Affiliations:** 1 Department of Physiology, All India Institute of Medical Sciences, Gorakhpur, Uttar Pradesh, India; 2 Department of Nursing, All India Institute of Medical Sciences, Gorakhpur, Uttar Pradesh, India; 3 Department of Community Medicine/Dean Research MRU, All India Institute of Medical Sciences, Gorakhpur, Uttar Pradesh, India; 4 Department of Nursing, All India Institute of Medical Sciences, Gorakhpur, Uttar Pradesh, India; 5 Department of Community Medicine, All India Institute of Medical Sciences, Gorakhpur, Uttar Pradesh, India; 6 Department of Physiology, All India Institute of Medical Sciences, Gorakhpur, Uttar Pradesh, India; 7 Department of Microbiology, All India Institute of Medical Sciences, Gorakhpur, Uttar Pradesh, India; 8 Multidisciplinary Research Unit, All India Institute of Medical Sciences, Gorakhpur, Uttar Pradesh, India

**Keywords:** Type 2 diabetes mellitus, autonomic dysfunction, yoga therapy, cardiovascular autonomic neuropathy, glycaemic control, lifestyle intervention

## Abstract

Type 2 diabetes mellitus (T2DM) is characterized by chronic hyperglycaemia, insulin resistance and β-cell dysfunction, leading to systemic inflammation and autonomic nervous system impairment. Diabetic autonomic neuropathy and cardiac autonomic neuropathy significantly increase risks of cardiovascular morbidity and mortality. Metabolic disturbances such as oxidative stress, polyol pathway activation and AGEs contribute to progressive neuronal and microvascular injury. Emerging evidence shows that yoga improves glycaemic control, autonomic balance and neuro-endocrine functioning in individuals with T2DM. Thus, we show that yoga as a complementary therapeutic approach to mitigate neuro-immunological dysregulation and enhance cardiovascular health in T2DM.

## Background:

T2DM is a chronic metabolic disorder characterized by elevated blood sugar levels resulting from insulin resistance and/or pancreatic β-cell dysfunction, affecting nearly every organ in the body. It develops due to consistently high blood glucose levels caused by insufficient insulin secretion from pancreatic β-cells or poor insulin usage. Various factors contribute to this condition, including unhealthy dietary patterns, physical inactivity, genetic predispositions and psychological stress, which can often impact future generations. Serious acute episodes of hypoglycemia or hyperglycemia can occur and may necessitate hospitalization [1]. T2DM is the most prevalent type of diabetes and is mainly associated with lifestyle choices. Insulin resistance causes prolonged hyperglycemia, which elevates the risk of cardiovascular events. It has been reported that about 425 million individuals globally had diabetes in 2017 and this figure is expected to rise to 629 million by 2045 [2]. The increasing rates of obesity, poor dietary habits and decreased physical activity have fuelled its rapid growth, making T2DM a major public health concern. Factors like obesity, poor diets and diminishing physical activity levels have contributed to its swift increase, establishing T2DM as a significant public health issue of the twenty-first century [3]. Proper dietary management and consistent physical activity are fundamental non-pharmacological strategies for managing T2DM along with other lifestyle-related conditions such as obesity, hypertension and dyslipidemia. Nevertheless, urbanization, calorie-rich diets, mechanized lifestyles, limited access to open spaces and a fast-paced modern routine hinder compliance with these strategies. Furthermore, individuals with T2DM often experience decreased physical activity due to obesity, a sedentary way of life, joint issues and diabetes-related complications such as cardiovascular diseases and neuropathy [4].

## Type 2 Diabetes Mellitus (T2DM) and the Immune System:

Around 90% of diabetes cases are included as Type 2 Diabetes Mellitus (T2DM). T2DM patients developed insulin resistance and impaired pancreatic β-cells that secrete insulin. In healthy physiology, elevated blood glucose stimulates pancreatic β-cells to release insulin, which binds to receptors on target cells, triggering the translocation of glucose transporter (GLUT) to the cell membrane and facilitating glucose uptake. In T2DM, insufficient insulin production or defective insulin signalling leads to glucose accumulation in the blood, resulting in chronic hyperglycaemia that progressively damages multiple organs and tissues [5]. Insulin resistance is closely linked to obesity, sedentary lifestyles and ageing. Initially, pancreatic islets attempt to compensate by increasing β-cell mass and insulin secretion. However, failure of this regulatory mechanism, β-cell function declines and many patients require insulin from outside, often within a decade of persistent insulin resistance. Prolonged hyperglycaemia contributes to both macrovascular complications like atherosclerosis and microvascular complications, including diabetic nephropathy, neuropathy and retinopathy. These complications affect the brain, heart, kidneys and eyes and significantly increase morbidity and mortality [6].

T2DM also increases susceptibility to infections, with affected individuals experiencing a higher incidence of pulmonary tuberculosis, pneumonia, urinary tract infections (UTI) and skin or soft tissue infections. These infections are likely more severe, less responsive to treatment and more costly due to extended hospitalisation [7]. Chronic inflammation is a key factor in the onset of insulin resistance. In cases of obesity, adipose tissue shows increased concentrations of tumour necrosis factor-alpha (TNF-α) along with other inflammatory agents such as interleukin-6 (IL-6), C-reactive protein and plasminogen activator inhibitor-1. Inflammatory substances like TNF-α, free fatty acids, diacylglycerol, ceramide, reactive oxygen species (ROS) and low oxygen levels activate IκB kinase β (IKKβ) and c-Jun N-terminal kinase 1 (JNK1) in both adipose and liver tissues. The activation of IKKβ and JNK1 facilitates the phosphorylation of serine residues on insulin receptor substrates (IRS-1 and IRS-2), which hinders their crucial tyrosine phosphorylation needed for proper insulin signaling. Additionally, TNF-α interferes with the function of peroxisome proliferator-activated receptor-gamma (PPAR-γ), exacerbating insulin resistance [8]. These pathways obstruct insulin's effectiveness and perpetuate systemic metabolic issues, particularly in individuals with obesity. The activation of IKKβ is essential for promoting insulin resistance associated with inflammation. It initiates the phosphorylation of IκB proteins, resulting in their degradation and the activation of nuclear factor κB transcription factors, which migrate to the nucleus and initiate the expression of various pro-inflammatory genes linked to immune reactions and chronic inflammation. Concurrently, IKKβ disrupts insulin signaling in adipocytes by serine phosphorylation of IRS-1, consequently impairing the insulin-stimulated glucose uptake process [9]. TNF-α activates c-Jun N-terminal kinase (JNK), contributes to insulin resistance by directly phosphorylating IRS-1 on serine residues, thereby interfering with its function in the insulin signalling cascade. The JAK/STAT pathway also negatively regulates insulin signaling. JAK kinases phosphorylate tyrosine residues of STAT proteins, causing them to dimerize and move to the nucleus, where they initiate gene transcription. This pathway plays a part in insulin resistance by causing serine phosphorylation of IRS-1 at Ser636 and Ser307, which results in the suppression of insulin signaling [10]. As a result, GLUT-4 translocation to the plasma membrane is impaired, thereby reducing glucose uptake and contributing to hyperglycaemia. The immune response characterized by inflammation, triggered by the death of fat cells and the infiltration of macrophages, is further intensified by pathogenic CD4+ and CD8+ T cells along with CD11c+ M1 macrophages present in adipose tissue of obese individuals, worsening both inflammation in adipose tissue and insulin resistance in the body. Consequently, pancreatic β cells increase insulin production in response to heightened insulin resistance, resulting in elevated levels of insulin in the blood. However, prolonged insulin resistance gradually exhausts β-cell functionality, leading to an insufficient supply of insulin. Additionally, the buildup of free fatty acids, amyloids and inflammatory cytokines promotes apoptosis of β cells, ultimately causing persistent high blood sugar levels and Type 2 Diabetes (T2DM) [11]. Figure 1 (see PDF) Illustration of disrupted insulin signalling induced by pro-inflammatory cytokines. In individuals with T2DM, the immune response is weakened. Elevated blood sugar and lack of insulin lead to various immune dysfunctions. Additionally, diabetic neuropathy can affect the integrity of natural barriers like the skin, increasing the likelihood of pathogen entry. T2DM is linked to irregularities in both innate and adaptive immune response, which include reduced cytokine production, impaired phagocytic activity, unregulated immune cell responses and a diminished capacity to eliminate microbial pathogens [12].

## Autonomic nervous system (ANS) and Type 2 Diabetes Mellitus:

The autonomic nervous system (ANS) plays a crucial role in maintaining homeostasis by controlling involuntary physiological processes through neural pathways and signaling molecules, including both sympathetic and parasympathetic. Together, these pathways control the vital processes such as heart rate, blood pressure, thermoregulation, urination and digestion [13]. In type 2 diabetes mellitus (T2DM), persistent metabolic disturbances frequently lead to autonomic dysfunction, known as diabetic autonomic neuropathy (DAN). Clinically, DAN may present with cardiovascular complications such as orthostatic hypotension, supine hypertension, reflex syncope and postural orthostatic tachycardia syndrome (POTS) [14]. Patients may also experience abnormal sweating patterns, including hyperhidrosis or hypohidrosis. The pathogenesis of DAN is multifactorial. Chronic hyperglycaemia and dyslipidaemia activate harmful biochemical pathways such as the polyol pathway, protein kinase C activation, oxidative stress and the formation of advanced glycation end-products (AGEs)-which damage nerve axons, Schwann cells and the microvascular endothelium. Elevated non-esterified fatty acids (NEFAs) undergo β-oxidation to generate toxic acylcarnitines, while oxidised LDL and AGE-receptor binding further intensify oxidative stress. Additionally, altered lipid metabolism may produce neurotoxic deoxy sphingolipids, contributing to nerve injury [15]. Autonomic neuropathy commonly occurs alongside peripheral neuropathy, particularly in individuals with T2DM and associated with vagus nerve dysfunction. Neuropathic changes typically affect distal sensory fibers, with axon loss and endoneurial microangiopathy impacting both large and small fibers. While microvascular injury, disease duration and sustained hyperglycaemia also play pivotal roles [16]. Early microvascular changes, detectable before clinical symptoms, may predict the future onset of diabetic neuropathy.

Pro-inflammatory cytokines such as TNF-α and IL-6 activate stress kinases (JNK1, IKKβ) and the JAK/STAT-3 pathway, leading to IRS-1 serine phosphorylation, impaired insulin signaling and reduced GLUT4 translocation. This cascade promotes chronic hyperglycemia, β-cell exhaustion and apoptosis, while NF-κB-mediated pro-inflammatory gene expression and reduced PPAR-γ activity further aggravate systemic insulin resistance and diabetic complications including neuropathy, nephropathy, retinopathy and cardiovascular dysfunction.

## Autonomic Dysfunction in Type 2 Diabetes Mellitus:

T2DM is the primary cause of neuropathy, which leads to various symptoms that affect multiple nerve types and are involved in different pathogenic processes, including ischemic, metabolic, compressive and immune-related mechanisms. Diabetic autonomic neuropathy (DAN) is often seen alongside diabetic sensorimotor polyneuropathy (DSP). During the initial condition, autonomic symptoms tend to be mild or subclinical, becoming more noticeable as the condition advances [17]. The specific mechanisms that contribute to DSP are not fully known. But its pathophysiology involves a complex interaction between glycemic management, age-related nerve degeneration, disease duration and other associated factors such as hypertension, dyslipidaemia and obesity. Chronic hyperglycemia, glucotoxicity and disrupted insulin signaling initiate various biochemical pathways that disturb cellular metabolism, leading to structural damage in nerves. These alterations involve Wallerian degeneration, segmental demyelination, microangiopathy and apoptosis of neurons in the dorsal root ganglia, which negatively affects myelination and contributes to the loss of nerve fibers [18]. Elevated glucose levels result in increased free radical production within mitochondria and in the absence of sufficient antioxidants, this triggers further harmful mechanisms. These harmful processes include an elevated generation of advanced glycation end-products (AGEs), a reduction in the soluble receptor for AGEs, activation of the polyol pathway via aldose reductase, protein kinase C accumulation, stimulation of cyclooxygenase-2, activation of poly (ADP-ribose) polymerase, endothelial cell dysfunction, production of peroxynitrite, protein nitration and alterations in Na^+^/K^+^-ATPase activity [19]. Collectively, these mechanisms impair neuronal communication, modify membrane permeability, harm the vascular supply to nerves and disrupt mitochondrial energy metabolism. The previous study reported that it reduced the nerve conduction velocity and prolonged terminal latency in patients with abnormal heart rate responses to the Valsalva manoeuvre. At the same time, cardiovascular autonomic dysfunction and peripheral neuropathy often coexist; their relationship is variable. Non-neural factors, such as increased carotid intima-media thickness, decreased arterial distensibility, baroreceptor dysfunction, left ventricular hypertrophy and endothelial impairment, may also contribute to autonomic disturbances. These abnormalities are strongly associated with microalbuminuria, which is regarded as a marker of endothelial damage in diabetes [20]. In both type 1 and type 2 diabetes, the autonomic dysfunction and microalbuminuria are linked to higher mortality risk. Conversely, autonomic dysfunction may worsen renal outcomes, creating a cycle of progressive organ injury. Studies have shown that the prevalence and severity of cardiovascular autonomic dysfunction are strongly correlated with microvascular complications, including retinopathy, nephropathy and erectile dysfunction [21]. However, the previous research findings suggested that cardiovascular autonomic dysfunction and microalbuminuria are related; neither independently predicts cardiovascular mortality, possibly due to methodological limitations in their scoring system. Another mechanism through which autonomic dysfunction may increase mortality is heightened susceptibility to lethal arrhythmias, similar to post-myocardial infarction patients. This is supported by the high proportion of unexplained sudden deaths in diabetic populations. Interestingly, recent studies suggested that prolonged hyperglycaemia is the sole cause of autonomic neuropathy, reporting dysautonomia even in non-diabetic individuals with metabolic syndrome features and microvascular injury [22]. Cardiac autonomic neuropathy (CAN) dysfunction, which includes both parasympathetic and sympathetic, is linked to myocardial ischemia, arrhythmias and sudden cardiac death. Typically, parasympathetic dysfunction is observed before sympathetic impairment, highlighting the importance of early detection. According to the American Diabetes Association, CAN can be assessed through five standardized cardiovascular reflex tests, including heart rate variability with deep breathing, the Valsalva manoeuvre, postural heart rate changes and blood pressure responses to standing and sustained handgrip. These tests evaluate end-organ responses and help localize autonomic deficits. CAN prevalence in diabetic populations has been reported to range from 20% to as high as 76%, depending on diagnostic criteria and patient selection [23]. Diabetic autonomic neuropathy (DAN) can occur in both Type 1 and Type 2 diabetes and is defined as a functional or structural impairment of sympathetic and/or parasympathetic pathways. Its manifestations vary depending on the organ system involved, with cardiovascular symptoms such as arrhythmias, orthostatic hypotension and exercise intolerance being most common. Risk factors for DAN include long diabetes duration, poor glycaemic control, advanced age and the presence of other microvascular complications such as neuropathy, nephropathy and retinopathy, as well as comorbid conditions like hypertension and dyslipidaemia [24]. The underlying pathogenesis of diabetic neuropathy involves multiple mechanisms, including oxidative stress, immune-mediated injury and accumulation of advanced glycation end-products (AGEs), nutritional deficiencies, neurovascular damage, hormonal imbalances and chronic metabolic insult to neurons [15].

## YOGA practices in type 2 diabetes:

Yoga originated from the ancient Indus Valley civilization, reflecting its deep roots in Indian culture. It serves as a holistic practice for physical, mental and spiritual well-being. Engaging in yoga can aid in the management of a variety of lifestyle-related diseases, such as type 2 diabetes. The positive impacts of yoga on diabetes are linked to psycho-neuro-endocrine and immune system mechanisms. Once viewed primarily as a spiritual discipline, yoga is now recognized globally as a holistic approach to health management rather than a disease-specific therapy [25]. Yoga is a multifaceted practice involving cleansing techniques (kriya), postures (asanas), controlled breathing (pranayama), meditation, relaxation, chanting, diet, ethical principles and spiritual philosophy. It also influences eating behaviours and has shown promise in managing eating disorders through enhanced self-awareness and mindfulness. Practices like pranayama and Sudarshan Kriya have been shown to improve dietary habits and medication adherence [26]. In the management of type 2 diabetes, several yoga techniques have proven beneficial. By addressing the person as a whole, including physical inactivity and unhealthy behaviours, yoga can be adapted in intensity and form to meet diverse clinical needs.

## Role of Yoga in the Management of Type 2 Diabetes Mellitus:

Yoga practice is based on the principle of an intrinsic connection between the mind and body. Consistent yoga practice increases body flexibility, muscle strength, blood flow and oxygen consumption. In chronic lifestyle disorders such as type 2 diabetes mellitus (T2DM), yoga fosters adherence to therapeutic regimens by promoting discipline in dietary habits and physical activity. T2DM significantly reduces quality of life and is often aggravated by psychological stress. It triggers the hypothalamic-pituitary-adrenal (HPA) axis and the sympathetic nervous system, leading to increased cortisol levels, catecholamines and pro-inflammatory cytokines and plays a significant role in the development of insulin resistance, impaired glycaemic control and issues like neuropathy. By alleviating stress, yoga facilitates both metabolic balance and mental health [27]. Clinical studies show yoga improves overall wellness, reduces depression and anxiety and enhances quality of life. Behaviourally, it encourages healthier exercise patterns and dietary choices. Physiologically, yoga postures may stimulate pancreatic cell regeneration, improve β-cell responsiveness to glucose and increase skeletal muscle glucose uptake. Hormonal balance achieved through yoga further aids glycaemic regulation [28]. Yoga also exerts immunomodulatory effects, reducing systemic inflammation and enhancing immune function. It increases endorphins, serotonin, dopamine and melatonin-neurochemicals linked to mood stability, stress reduction and emotional balance. Previous studies suggested that yoga may mobilize stem cells from bone marrow to peripheral tissues, promoting tissue repair, although further research is needed [29]. Beyond immune modulation, yoga influences gene expression, muscle performance, endurance, flexibility and balance. These adaptations improve body composition, reduce adiposity, optimize lipid profiles and enhance insulin sensitivity. It also lowers oxidative stress markers such as malondialdehyde and interleukin-6 (IL-6) while increasing adiponectin, an anti-inflammatory adipokine. Pulmonary function and immune parameters, including lymphocyte migration, have shown measurable improvement in T2DM patients [30]. Engaging in asana and pranayama may provide better glycaemic management than standard lifestyle recommendations because of their increasing intensity and length. These practices promote muscle contractions and facilitate the movement of glucose transporters, which leads to improved insulin sensitivity. Interestingly, those with lower baseline glycaemic control seem to receive the greatest advantages [31]. Yoga's positive effects extend to lipid metabolism, with documented reductions in total cholesterol and triglycerides, alongside improved HDL levels. This is particularly important as dyslipidaemia in diabetes heightens cardiovascular risk. The metabolic effects of yoga may parallel those of sustained endurance exercise [32]. Evidence supports yoga's role in lowering fasting and postprandial glucose, HbA1c and the need for anti-diabetic medications. It promotes reductions in body weight gain, BMI, waist to hip ratio and fat mass while preserving lean mass. Lipid improvements-including reduced triglycerides, LDL and free fatty acids and increased HDL-are consistently reported. Long-term practice has also been linked to better insulin sensitivity and lower insulin resistance in both diabetic and healthy individuals [33]. The Integrated Approach to Yoga Therapy (IAYT), combining asanas, pranayama, kriyas, meditation and yogic education, has been shown to reduce plasma glucose level, HbA1c, cholesterol level and medication dependency in T2DM patients [34]. Yoga enhances cardiovascular fitness, maintains systolic and diastolic blood pressure and improves cardiac autonomic function, by reducing the risk of arrhythmias and sudden cardiac death. Other benefits include improved nerve conduction, cognitive performance and coagulation stability. Yoga improves glycaemic condition without increasing body weight and may promote gradual weight loss. Its safety, accessibility, low cost and multi-system benefits make it a complementary therapy for both prevention and management of T2DM.

## Specific yogic practices beneficial in Type 2 diabetes mellitus:

Yoga offers a multifaceted approach to managing T2DM through its diverse set of practices that target physiological and neuro-immunological domains. Practices like Surya Namaskar (Sun Salutation) stimulate insulin production by engaging the endocrine system through dynamic movement. Specific hasta mudras such as Apan mudra, Gyan mudra, Linga mudra and Surya mudra promote deep relaxation, enhance metabolic rate, support weight loss and reduce symptoms like sugar-induced vomiting by lowering circulating free fatty acids. Cleansing practices like Shankhaprakshalana (intestinal cleansing) aid in increasing insulin production and lowering blood glucose levels by improving gut health. Breathing techniques or pranayama, including Anulom Vilom, improve cardiorespiratory fitness, while Bhramari pranayama is a breathing exercise of yoga in which you make a humming sound from the throat, which exerts a calming effect on the mind. Uddiyan Bandha (abdominal lock) is practiced by pulling the abdomen inwards after exhalation and holding the breath outside, which improves pancreatic function and Surya Bhedan (right nostril breathing) enhances sympathetic activity, making it beneficial in diabetes management. Chanting "Aum" stabilizes the brain and boosts energy levels and Yoga Nidra significantly reduces fasting and postprandial blood glucose levels by promoting deep relaxation [35]. In terms of asana practice, forward bends, backward bends and inversions rejuvenate pancreatic cells through alternating abdominal compression and relaxation, enhancing insulin receptor sensitivity in muscle tissues and stimulating insulin secretion. These postures also promote blood circulation and exert both stimulating and energizing effects. Meditation positively controls blood sugar levels by reducing stress and improving emotional regulation. Shuddhi kriyas like Kapalbhati pranayama generate abdominal pressure that enhances the efficiency of pancreatic β-cells. Mindfulness practices contribute to better sleep and deeper relaxation, indirectly supporting glycaemic control. Agnisar kriya, involving rapid abdominal movements, creates vacuum effects that relax the internal organs and increases blood supply to the abdominal region. Vaman Dhauti (stomach cleansing) [36] enhances glucose uptake, reduces insulin resistance and supports overall insulin function. Together, these yogic practices serve as a powerful, adjunct in the holistic management of T2DM.

## Yoga Therapy improves autonomous function in T2DM:

Hyperglycaemic condition triggers oxidative stress and promote the formation of advanced glycation end products (AGEs). These pathological changes damage autonomic neurons through increased inflammatory cytokine production, reduced nitric oxide bioavailability and impaired endothelial function. Mitochondrial dysfunction and altered neurotransmitter levels, such as decreased acetylcholine and norepinephrine, further contribute to autonomic imbalance and disrupted neural signalling [15]. Yoga has emerged as a promising adjunctive therapy in T2DM that improves glycaemic control by modulating autonomic nervous system activity. Yoga enhances mental well-being and may reduce diabetes-related complications. Evidence from recent studies reinforces its therapeutic value: a 2020 study demonstrated that holistic lifestyle changes incorporating yoga improved both autonomic balance and glycaemic control in T2DM patients [37]. Previous studies reported that regular yoga practice may result in significant improvements in heart rate variability (HRV), stress biomarkers and psychological well-being, while a clinical study reported that cardiovascular benefits through enhanced autonomic regulation, highlighting yoga's role in reducing stress-related diabetic complications [38]. Autonomic function test (AFT) data further support these observations. T2DM patients commonly exhibit reduced heart rate responses to standing, Valsalva manoeuvre and deep breathing, indicating impaired parasympathetic activity that exaggerated blood pressure responses to cold pressor, handgrip and standing tests, reflecting sympathetic overactivity. Conversely, yoga practitioners show higher HRV and lower blood pressure reactivity, suggesting better autonomic balance [39]. These improvements are clinically significant, as autonomic dysfunction in diabetes is strongly linked to increased cardiovascular risk, arrhythmias and sudden cardiac death. By promoting parasympathetic dominance and reducing sympathetic hyperactivity, regular yoga practice may help mitigate these risks. Yoga helps to diminish oxidative stress and inflammation, which are significant factors in autonomic neuropathy, by boosting the activity of antioxidant enzymes, reducing reactive oxygen species (ROS) and lowering levels of pro-inflammatory cytokines like IL-6 and TNF-α. Additionally, it promotes the expression of brain-derived neurotrophic factor (BDNF), which aids in the growth and survival of neurons. Additionally, regular yoga practice controls the hypothalamic-pituitary-adrenal (HPA) axis, which is crucial for stress management, lowering cortisol levels and further promoting parasympathetic activation [40]. Through its combined effects on metabolic control, oxidative stress reduction and neural regulation, yoga represents a safe and effective complementary therapy for improving autonomic function and reducing cardiovascular risk in T2DM.

## Conclusion:

Yoga offers a valuable complementary approach for managing type 2diabetes by improving glycaemic control, reducing inflammation and restoring autonomic balance. Yoga helps lower cardiovascular risk and enhances metabolic stability through its physiological and neuro-immunological benefits. Incorporating regular yoga practice can significantly reduce complications, support medication efficiency and improve overall quality of life in individuals with T2DM.

## Author's contribution:

Concept and design: AMD, CR, MKS, RK, VR

## Literature search:

MKS, SN, GM

## Manuscript preparation and Editing:

MKS, CR, GM, AR, VR

## Critical review and technical inputs:

MKS, AR

## Final approval of the manuscript:

All authors
